# The effect of cognitive ability on academic achievement: The mediating role of self-discipline and the moderating role of planning

**DOI:** 10.3389/fpsyg.2022.1014655

**Published:** 2022-10-06

**Authors:** Yueqi Shi, Shaowei Qu

**Affiliations:** School of Humanities and Social Sciences, University of Science and Technology Beijing, Beijing, China

**Keywords:** cognitive ability, self-discipline, planning, academic achievement, structural equation modeling

## Abstract

In this study, 572 secondary school students aged 15–18 years old stage were selected to study the effect of their cognitive ability and self-discipline and planning on academic achievement. Cognitive ability was classified into memory ability, representational ability, information processing ability, logical reasoning ability, and thinking conversion ability, and analyzed the effects of these five ability values on academic achievement. The mediating effect of self-discipline ability between cognitive ability and academic achievement was analyzed using structural equation modeling (SEM), and the moderating role of planning in the mediating effect was analyzed using planning as a moderating variable. The results showed that cognitive ability can have a significant positive effect on academic achievement, while self-discipline plays a partially mediating role between cognitive ability and academic achievement, and the moderating effect of Planning is significant in the second half of the mediating effect, i.e., the effect of self-discipline on academic achievement changes as the level of planning increases, and the mediating effect is stronger in the condition of higher planning, and the mediating model with moderating effect holds.

## Introduction

According to Zhao (2017), academic achievement refers to the actual performance of students’ mastery of academic knowledge and skills as demonstrated through examinations after systematic knowledge and skills learning. In the Chinese educational student evaluation system, universities usually classify students by academic achievement. Liu (2019) argued that academic achievement, especially in China’s college entrance exams, determines students’ future development, so studying the factors that enhance academic achievement will help each student’s learning and development. Under the educational selection system that is being implemented in China, academic achievement is the measure of students’ academic achievement and the most important reference factor for admission to colleges and universities.

Both cognitive ability and self-discipline have been key factors affecting students’ academic achievement (Liang et al., 2020). Self-discipline has become one of the key factors governing students’ academic achievement, especially since schools adopted online instruction following the outbreak of COVID-19 (Schulz, 2021). However, it is not clear the way in which cognitive ability and self-discipline work together to influence students’ overall academic achievement. Also, planning, an important factor influencing academic achievement (Cao and Cao, 2004), has rarely been examined alongside cognitive ability and self-discipline for its impact on academic achievement.

In this study, the cognitive ability, self-discipline, planning, and academic achievement of high school students were studied, and a structural equation with a moderating mediating effect was constructed with self-discipline as the mediating variable and planning as the moderating variable to analyze the mediating effect of self-discipline between cognitive ability and academic achievement, and the moderating effect of planning under the mediating effect.

### The effect of cognitive ability

Cognitive ability refers to the human brain’s ability to store memory, process and extraction of information, includes attention, memory and logical reasoning, and thinking transformation. It is a key factor that research can consistently predict Academic Achievement (Stadler et al., 2016). Past research has centered on the direct effect of personal decent cognitive abilities of students on Academic Achievement (Kuncel et al., 2004; Miriam et al., 2011). Rohde and Thompson (2007) concluded that cognitive ability can directly affect academic achievement with a correlation of 0.38. Ian et al. (2006), in a study of over 70,000 British students A 5-year follow-up study found a correlation between cognitive ability and academic achievement of 0.81. Grass step-by-step analysis through multivariate analysis obtained that logical reasoning skills can significantly affect students’ performance in science and chemistry (Grass et al., 2017). Liu et al. (2021) measured the cognitive abilities of spatial imagination, computation, and information processing in 499 Chinese children and teamed up to analyze the association between students’ academic achievement in mathematics and Chinese for two consecutive school years and found significant correlations between visual-spatial imagination, computation, and information processing abilities and academic achievement. Most such previous studies have examined the single effect of cognitive ability on academic achievement at the individual student level (Kuncel et al., 2004; Miriam et al., 2011). In addition, the above findings support the knowledge process theory (Deary et al., 2006; Xu and Li, 2015), which concludes that when students’ cognitive abilities are high, they are able to encode key information more quickly and accurately in memory, thus enabling the brain to output more and more effective information, resulting in better academic achievement on exams (Liu and Wang, 2000; Zhang and Zhang, 2011). Conversely, at lower levels of cognitive ability, some knowledge is missed in the knowledge process, which further reduces effective information output and leads to lower academic achievement (Miriam et al., 2011). These findings also support the results of previous analyses indicating that cognitive ability usually contributes significantly to academic achievement.

Although there is a significant relationship between cognitive abilities and Academic Achievement, existing research is difficult to model the effect of cognitive abilities on Academic Achievement. In fact, in terms of student learning, cognitive abilities are very important in students’ learning activities, and it is not only about different cognitive abilities, but also relates to the ways in which these different abilities function together (David, 2005). There are many controversies about the pattern of influence of cognitive ability on Academic Achievement in different studies (Formazin et al., 2011). Zhang (2008) found that logical reasoning ability (LRA) had a correlation coefficient of about 0.3 with Chinese and mathematics scores, while thinking transformation ability (TCA) had no significant correlation with scores in these two subjects. However, Xu and Li (2015) found a significant correlation between thinking transformation ability and performance in these two subjects. These results suggest that the complex role of cognitive ability is difficult to reveal comprehensively and systematically when only the effect of a single cognitive factor on Academic Achievement is examined.

Through the research of many scholars, we can find that the effect of students’ cognitive ability on academic achievement is significant, but the complex mechanisms of their influence remain very ambiguous. The importance of cognitive abilities in students’ learning activities is only reflected in the researcher’s predetermined scope of investigation, which contains one or more cognitive abilities specific to the researcher, while outside the scope of investigation, these cognitive abilities still operate in an unpredictable manner (David, 2005), thus, scholars still do not reach a consensus on why cognitive abilities affect academic achievement due to the inconsistent scope of investigation of students’ cognitive abilities (Formazin et al., 2011).

In addition, Past research has tended to examine the impact of a single cognitive ability, while research under the combined influence of multiple cognitive abilities is lacking. Therefore, in this study, according to classification of cognitive abilities by Wo and Lin (2000) and Liang et al. (2020), these researchers conducted numerous studies and explored five categories of cognitive abilities, namely, information processing, logical reasoning, working memory, thought transformation, and representation, getting scientific and valid conclusions and summarizing the test methods for different cognitive abilities. Specifically, they developed a software system to measure these five cognitive abilities, which has measured more than 2 million students in mainland China, and obtained normative data applicable to measure the cognitive ability of students in mainland China at this stage. This cognitive ability assessment system has been fully tested for its reliability and validity, and can assess students’ cognitive ability very accurately. In addition, the Chinese invention patent of this cognitive ability assessment system has been obtained by Wo (2010). Therefore, this study explored the specific effects of different cognitive abilities on academic achievement and proposed the following hypotheses.

**Hypothesis 1:** Cognitive ability can positively predict students’ academic achievement.

### The mediating role of self-discipline

Many studies are generally agreed by researchers that cognitively competent students have better Academic Achievement (Kuncel et al., 2004; Miriam et al., 2011; Stadler et al., 2016). However, there are several other researchers’ studies that suggest that cognitive ability is only one of many determinants of high academic achievement of students (Shao, 1983). Several studies find that self-discipline is also a factor that influences how well students achieve academically, and self-discipline is the ability of students to manage their self-perceptions, emotions, and behaviors consciously according to learning requirements or their own goal expectations without external supervision or restrictions (Xie, 2009). Dai (2013) examined the correlation between self-regulation, emotional stability, and academic achievement and found a significant positive relationship between self-discipline and academic achievement. Duckworth and Seligman (2010) used American eighth-grade students as studied the effect of self-regulation on academic achievement and found that self-regulation had a positive effect on student achievement. Zhao (2017) found that self-discipline significantly predicted academic achievement in upper elementary school students. Wang (2003) designed his own self-discipline questionnaire and surveyed 885 middle to high school students in Shanghai and showed that self-discipline was a significant positive predictor of academic achievement. Many previous studies have shown a significant positive correlation between students’ self-discipline and academic achievement. However, the mechanism by which self-discipline affects academic achievement remains unclear.

Cognitive ability and self-discipline are both independent and interrelated elements of individual students’ psychology (Li and Zhang, 2015), since cognitive ability and self-discipline are indicators of different dimensions of students in the learning process, many scholars have different opinions about their relevance (James et al., 2006). Most studies have concluded that the correlation between self-discipline and cognitive ability is small, and therefore, cognitive ability and self-discipline are often used as independent variables affecting students’ academic achievement (Roemer et al., 2022); however, some scholars have argued that cognitive ability and self-discipline influence each other (Borghans et al., 2008), and Ruffing, in conducting an analysis of factors influencing academic achievement, found that both cognitive ability and self-discipline can significantly and positively influence academic achievement, and that cognitive ability and self-discipline have a correlation (Ruffing et al., 2015).

In recent years, the correlation relationship between cognitive ability and self-discipline has received more attention, and some researchers have used long-term follow-up survey data to confirm the influence relationship between cognitive ability and self-discipline both theoretically and empirically, and found that cognitive ability can significantly influence self-discipline (Heckman et al., 2018). Self-discipline enables students to focus more on tasks and achieve better academic achievement (Nesayan et al., 2018). Therefore, the following hypotheses were formulated in this study.

**Hypothesis 2:** Self-discipline mediates the relationship between cognitive ability and academic achievement.

### Moderating effect of planning

Planning is the psychological and behavioral characteristics of an individual’s use of time and has a multidimensional and multilevel psychological structure (Zhang et al., 2001). Planning can directly affect students’ motivation, effort, thought processes and mental processes. Wang (2007) found that planning has a significant effect on the academic achievement of secondary school students. Good planning can better achieve the balance between wanting to learn and personality (Mirzaei et al., 2012), control and arrange their study and life rationally, and improve academic achievement (Li et al., 2016). Cao and Cao (2004) found that the ability to manage and organize time effectively greatly influenced the academic achievement of high school students through a study of their planning. Further research on the relationship between planning and students’ academic achievement found that planning had some predictive power on academic achievement, but it was only modeled from a single-factor perspective, without further research on multi-factor modeling (Zhang et al., 2001).

After considering cognitive abilities and personality traits, it was found that planning does not directly affect academic achievement, but rather acts as a moderator between academic achievement. Claessens et al. (2010) argued that although planning is related to academic achievement, the extent and form of its effect is not in a direct manner. Meanwhile, Claessens found, after a study, that planning, although it can generate a sense of control over time, only moderates academic achievement, because planning was related to factors such as cognitive variables and personality differences, the study found that there was no significant positive relationship between planning and academic achievement (Claessens et al., 2007). In addition, Macan (1994) proposed a time management process model based on an emphasis on task execution mechanisms (time planning, scheduling) and learning goals, and argued that planning as a latent variable moderates learning attitudes within learners.

In terms of self-discipline theory, planning regulates the implementation of individual goals and tasks, and effort management, including perseverance in performing tasks and self-motivation, ensures that goals are accomplished. Malte et al. (2009) also suggest that students regulate their own efforts through planning to achieve better performance. Studies have found that planning is a personality disposition that reflects or regulates cognitive activity (Dickson, 1981) and is a regulation of cognition. Cognitive activities can directly enable cognitive subjects to make progress in cognitive activities; whereas planning can only indirectly make progress in cognitive activities through the regulation of cognitive activities (Wang, 1999). Meanwhile, cognitive theory suggests that cognitive processes determine the production of emotions and behaviors, and changes in emotions and behaviors can also affect cognitive changes (Li, 1999). Based on the above findings, it is hypothesized that planning moderates the effect of cognitive ability on academic achievement when it affects academic achievement.

In addition, laxity in planning is the most obvious external manifestation of academic procrastination (Wu et al., 2014), which is often caused by individuals’ inability to control their own behavior and their inability to plan their time rationally, thus leading to lower learning efficiency and affecting academic achievement. Self-discipline is a stable and reliable personality trait, but self-discipline is a manifestation of students’ internal self-discipline, which cannot be mapped to the external study plan, and the lack of a scientific and reasonable study plan can also lead to academic procrastination and reduce study efficiency. For example, Fang and Wang (2003) found that there was no significant difference in self-discipline between students with good academic achievement and students with poor academic achievement, but there were significant or highly significant differences in five dimensions of time management: conceptual, planning, strategic, integrated, and immediate. Based on the above studies, it is hypothesized that planning moderates the effect of self-discipline on academic achievement when it affects academic achievement. It is hypothesized that planning moderates the effect of self-discipline on academic achievement.

Therefore, this study hypothesizes that self-discipline mediates between cognitive ability and academic achievement, and that planning moderates the mediating effect of self-discipline between cognitive ability and academic achievement, and proposes the following research hypothesis.

**Hypothesis 3:** planning can positively moderate the effect of cognitive ability on self-discipline.

**Hypothesis 4:** Planning can positively regulate the effect of self-discipline on academic achievement.

**Hypothesis 5:** planning can moderate the mediating role of self-discipline ability between cognitive ability and academic achievement.

**FIGURE 1:**
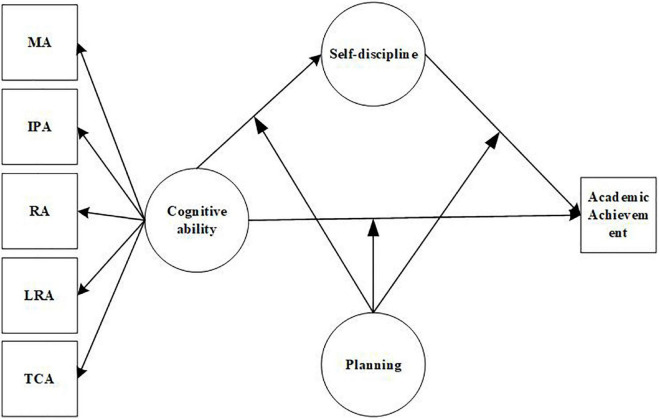
Structural equation relationship diagram. MA, Memory ability; IPA, Information processing ability; RA, Representation ability; LRA, Logical reasoning ability; TCA, Thinking conversion ability.

The main research relationships of the structural equation model are shown in Figure 1.

## Materials and methods

### Participants

The ethical examination of this study was approved by the Research Ethics Committee of the School of Humanities and Social Sciences, University of Science and Technology Beijing, and the study was conducted in accordance with the regulations for the protection of human subjects. This study selected 572 students as samples, all aged 15–18 years old, As shown in Table 1.

**TABLE 1:** Distribution of participating students.

Grade	Number of students			
	Boys	Proportion	Girls	Proportion
First grade	115	51.11%	110	48.89%
Second grade	83	46.63%	95	53.37%
Third grade	93	55.03%	76	44.97%
Total	291	50.87%	281	49.23%

### Procedure

Both the cognitive ability, Self-discipline and Planning measures in this research were conducted on campus. The students who took the test were organized by staff and tested in a separate classroom. The entire test lasted for 2 h.

Structural equation models were developed based on the obtained students’ cognitive ability, self-discipline, planning ability, and academic achievement, and composite achievement models and sub-subject models were developed for Chinese, mathematics, and English, respectively. Each model was first tested for common method bias in the analysis process, and then the fit of the model was tested according to the CFA test procedure. When all tests were passed, the mediating effect analysis process with moderation was followed by first bringing cognitive ability, self-discipline and planning ability into the structural equation model to see the model fit, then bringing the interaction term of planning on cognitive ability and self-discipline ability into the model to compare whether the model fit was optimized, and finally testing the mediating effect of self-discipline ability with the bootstrap method and analyzing the planning with the simple slope test moderation effect.

### Measures

#### Cognitive ability

A stimulus-informed cognitive ability system designed by Wo (2010) was used for the cognitive ability test. The stimulus information cognitive ability value test system adopts the world’s leading EEG ultra-low frequency fluctuation analyzer and ASL504/501 eye movement instrument as the basic research means, from the brain mechanism of individual psychological development, combining laboratory experiments and field experiments, using subtractive reaction time and additive reaction time (accurate to nanoseconds), microgenetics and other techniques, and adopting the form of microcomputer manual operation. The discriminative power and accuracy of the test results were greatly improved, and the accumulated sample norms of more than 2 million people were used to make the individual quantitative indicators of the subjects comparable with their peers.

The test questions of information processing ability include three parts: image selection response, graphic comparison response, and image matching response. Test questions for memory ability included two parts: Number forward and reverse memory and image matching response. Test questions of expressive ability include three parts: Spatial image manipulation, spatial image reasoning, and spatial image comparison. The test question of thought conversion ability is text-image matching test. The test questions for logical reasoning ability are conceptual reasoning and logical method reasoning.

The cognitive accuracy of the tested students was obtained by statistical methods, and their corresponding cognitive ability values were quantified and the quantified values were converted into T-scores to obtain the final cognitive ability values of the tested students. The final cognitive ability values contain five ability values: MA, LRA, RA, IPA, and TCA. The test method has been patented as an invention, and the sample size of the general test exceeds 2 million. The values of students’ cognitive abilities obtained from the test were normally distributed with a range of trends of ± 50 centered at 100, with high discriminant validity. The Cronbach’s alpha of the test ranged among 0.80–0.90.

#### Self-discipline

This study used the Self-discipline Scale designed by Zhang et al. (2005) for secondary school students. The scale has six questions on self-discipline. The positive questions were evaluated on a 5-point Likert scale: 5 (very much the same), 4 (comparatively the same), 3 (uncertain), 2 (relatively different), and1 (very different)—this test will test the score of each item. For example, the scores for each item of self-discipline were recorded as A1 to A6, where A4 was the reverse-scored questions. The total score for self-control was then A = (A1 + A2 + …… + 6)/6. The calculated total score is then converted into a Z-score based on the mean and standard deviation, and then the Z-score is converted into a T-score with a mean of 50 and a standard deviation of 10, then the T-score is the value of the test student’s self-discipline ability. The Cronbach’s alpha coefficients for the dimensions ranged from 0.60 to 0.93, with a validity of 0.91 and a test regression reliability of 0.85.

#### Planning

The planning scale was designed by Huang and Zhang (2001). The scale uses a 5-point Likert scale: 5 (very much the same), 4 (comparatively the same), 3 (uncertain), 2 (relatively different), and1 (very different). There were 24 questions. After assessing the students, the corresponding planning values were obtained by accumulating the scores of each question and converting them into T-scores as the values of students’ planning. The Cronbach’s alpha coefficients for the dimensions was 0.88.

#### Academic achievement

In the current research, in order to reduce the influence due to the level of students’ test performance, the average of the students’ four test scores in the semester when the cognitive ability was tested to be worthy was used as the academic score for each subject, and the raw scores were standardized (scores were assigned according to levels, with the highest score being 100 and the lowest being 0. In this study, three subjects, Chinese, Math and English, were selected for the study, and the composite academic score was the sum of the three subject’s total scores.

### Data analysis

This study first analyzed the correlations between cognitive ability, Self-disciplines, planning, and academic achievement, and then analyzed the mediating role of s self-disciplines and the moderating effect of planning using structural equation modeling according to the moderated mediating utility modeling procedure proposed by Wen and Ye (2014), and analyzed the pattern of the moderating role through a simple slope test. SPSS 25.0 and Mplus 8.3 software were used to analyze the data.

## Results

### Common method deviation test

The Harman one-way test was used to statistically control for common method bias, and the items of all variables were subjected to unrotated principal component factor analysis (Podsakoff et al., 2003), and an exploratory factor analysis of three variables (cognitive ability, Self-disciplines and planning) was conducted. According to the results, the three factors had eigenroots greater than 1 after factor rotation, with the first factor explaining 29.74% of the variance (less than 40% of the critical value) (Wang et al., 2016).

### Descriptive and bivariate analyses

The structural equation modeling was used in this study to examine the effects of cognitive ability, self-discipline, and planning on academic achievement, and the results are shown in Table 2. Cognitive ability, Self-disciplines, planning, and academic achievement all showed significant positive correlations. For more data, see Supplementary material.

**TABLE 2:** Means, standard deviations, and intercorrelations for variables.

	M	SD	1	2	3	4	5	6	7	8	9	10	11
1.MA	106.5751748	13.39039096	1										
2.IPA	105.1678322	11.39636837	0.478**	1									
3.RA	107.9003497	6.811872803	0.431**	0.463**	1								
4.LRA	106.1153846	7.937186042	0.382**	0.429**	0.302**	1							
5.TCA	97.3548951	14.69771038	0.484**	0.590**	0.516**	0.483**	1						
6.PLANING	103.3741259	14.72003395	0.217*	0.227*	0.066	0.012	0.158*	1					
7.SELFCONTROL	107.1748252	14.27037625	0.424**	0.488**	0.220**	0.359**	0.421**	0.096	1				
8.CHINESE	57.81797666	25.72765725	0.431**	0.489**	0.298**	0.377**	0.456**	0.578**	0.560**	1			
9.MATHEMATICS	50.47993002	28.91223569	0.390**	0.474**	0.257**	0.352**	0.430**	0.499**	0.568**	0.082	1		
10.ENGLISH	52.20492511	28.08333079	0.387**	0.389**	0.171**	0.350**	0.384**	0.527**	0.549**	0.300*	0.028	1	
11.TS	160.5028318	58.07603715	0.574**	0.644**	0.344**	0.513**	0.604**	0.562**	0.599**	0.531**	0.689**	0.681**	1

N = 572, *p < 0.05,**p < 0.001.

### Measurement model check

An exploratory factor analysis was required to check the quality of the model before conducting a moderated mediated effects analysis. This study included one latent variable, namely cognitive ability, and two explicit variables, self-discipline and planning. The results of the test showed a good fit of the model with χ^2^ (7) = 12.092, CFI = 0.992, TLI = 0.983, SRMR = 0.018, RMESA = 0.050, 90% CI = [0.013, 0.086], indicating that the fitted indicators were within the normal reception range. Table 3 also shows that the latent variable indicators have significant standardized loadings on the corresponding factors (*p* < 0.001).

**TABLE 3:** Factor loading coefficient table.

Variable	Non-std (coef.)	SD	*z* (CR)	Std
***Cognitive ability***				
MA	1	–	0.809003021	0.631***
IPA	0.999	0.073		0.741***
RA	0.508	0.042		0.631***
LRA	0.541	0.048		0.576***
TCA	1.383	0.101		0.796***

***p < 0.001.

### Moderating model checking

**FIGURE 2:**
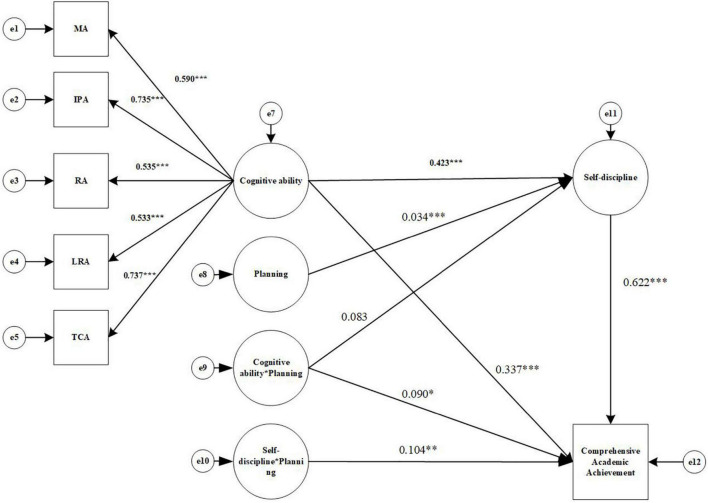
Structural equation modeling results diagram (Comprehensive Academic Achievement). MA, Memory ability; IPA, Information processing ability; RA, Representation ability; LRA, Logical reasoning ability; TCA, Thinking conversion ability. *p < 0.05, ^**^p < 0.01, ^***^p < 0.001.

In this study, structural equation modeling was used to examine the effects of cognitive ability, self-discipline, and planning on academic achievement, and the results are shown in Figure 2. The effects of cognitive ability on academic achievement, the mediating role of self-discipline, and the moderating role of planning were analyzed according to the mediated modeling process with regulation proposed by Wen and Ye (2014).

#### Model 1: Impact on comprehensive academic achievement

Referring to the steps of analysis of mediated model with moderation summarized by Wen and Ye (2014) and combined with the hypotheses of this study, Cognitive ability (X), Self-discipline (W), Comprehensive Academic achievement (Y), and Planning (U) were used as variables. In addition, all indicators were standardized to reduce the effect of multicollinearity. First, a direct effect model was developed with Cognitive ability(X) as the independent variable and Comprehensive Academic achievement(Y) as the dependent variable, and the direct effect was tested to see if it was moderated by Planning(U). The results show that the model without the interaction term fits well: χ^2^/df = 4.71, CFI = 0.942, TLI = 0.909, RMESA = 0.119, and SRMR = 0.065. For the mediated model with the interaction term, AIC = 73948.290, compared to the AIC value of the baseline model (74672.583), which is reduced by 724.293, indicating that the mediated model with adjustment has improved compared with the baseline model; At the same time, For the mediated model with the interaction term, Log Likelihood = –36931.145, which is 370.147 higher than the Log Likelihood of the baseline model (–37301.292), that is, the value of –2LL is 370.147, the degree of freedom of the model is increased by 8, and the chi-square test of –2LL value is significant (*p* < 0.05), so the mediated model with adjustment fits better than the baseline model.

Cognitive ability was a positive predictor of Comprehensive Academic achievement (β = 0.612, *p* < 0.001), 95% CI = [0.532, 0.681]; the interaction term of Cognitive ability and Planning was a significant predictor of Comprehensive Academic achievement (β = 0.144, *p* < 0.01), 95% CI = [0.064, 0.226]. It indicates that the direct relationship between Cognitive ability and Comprehensive Academic achievement is moderated by Planning.

Second, the effect of Cognitive ability (X) on Comprehensive Academic achievement (Y) through Self-discipline (W) was tested to see if it was moderated by Planning (U), and the results are shown in Figure 2. The data show that the model fit index is well: χ^2^/df = 5.15, CFI = 0.935, TLI = 0.899, RMESA = 0.126, and SRMR = 0.073. For the mediated model with adjustment including the interaction term, AIC = 65973.542, compared to the AIC value of the baseline model (74533.415), which is reduced by 8559.873, indicating an improvement of the mediated model with adjustment compared to the baseline model; meanwhile, the Log Likelihood of the mediated model with adjustment = –32943.771, compared to the Log Likelihood of the baseline model (–37230.708), increased by 4286.937, which means that the –2LL value is 4286.937. The increase in the degrees of freedom of the model is 8, and the chi-square test for the –2LL value is significant (*p* < 0.05), so the mediated model with adjustment fits better than the baseline model.

Cognitive ability (X) significantly predicts comprehensive Academic Achievement (Y) (β = 0.337, *p* < 0.001), 95% CI = [0.251, 0.420]. Cognitive ability (X) can significantly predict Self-discipline (W) (a1 = 0.423, *p* < 0.001), 95% CI = [0.340, 0.497]. The interaction term (UX) of Cognitive ability (X) and Planning (U) cannot significantly predict Self-discipline (W) (a3 = 0.083, *p* = 0.218), 95% CI = [–0.052, 0.210], containing 0. Self-discipline(W) can significantly predict Comprehensive Academic achievement(Y) (b1 = 0.622, *p* < 0.001), 95% CI = [0.550, 0.689]; the interaction term (UX) of Cognitive ability (X) and Planning (U) can significantly predict Comprehensive Academic achievement (Y) (c3′ = 0.090, *p* < 0.05), 95% CI = [0.007, 0.170]; the interaction term (UW) between Self-discipline (W) and Planning (U) can significantly predict Comprehensive Academic achievement (Y) (b2 = 0.104, *p* < 0.05), 95% CI = [0.034, 0.191]. In addition, 95% CI for a1b2, a3b1, and a3b2 were calculated using the Bootstrap method and were [0.016, 0.078], [–0.014, 0.050], and [0.000, 0.001], respectively, with only 95% CI for a1b2 not containing 0. Thus, it is evident that the Self-discipline in the mediating effect between cognitive ability and comprehensive academic achievement was significant (mediating effect = 2.884, SE = 0.398, *p* < 0.001, 95% CI = [2.148, 3.706]), while the discipline was moderated by Planning in the second half of the mediating effect, the effect of Self-discipline on comprehensive academic achievement varied with the level of planning; the first half of the mediating effect of Self-discipline was not moderated by planning. Hypotheses 2 and 4 are valid, but hypothesis 3 is not.

**FIGURE 3:**
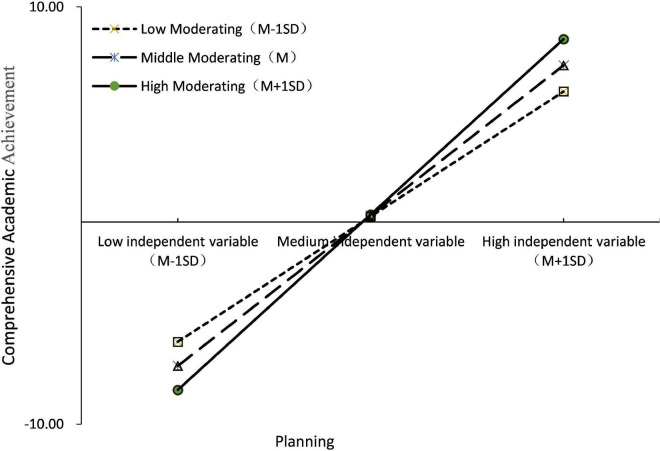
Simple slope test (comprehensive academic achievement).

To further explore the moderating effect of Planning, students were divided into “high/low Planning groups” using a positive/negative one standard deviation boundary for Planning. The simple slope test (see Figure 3) showed that Self-discipline was a significant positive predictor of Comprehensive Academic achievement in the “high Planning group” (β = 1.874, *p* < 0.001); in the “Low Planning group,” although the predictive effect was still significant (β = 1.846, *p* < 0.001), it was weaker than in the “High Planning group.” In addition, when the process of mediation is moderated, it is required to test whether the mediating effect varies with changes in the moderating variable U, as suggested by Edwards and Lambert (2007), which is to take the value of one standard deviation above and below the mean of U (standard UH = 1, UL =–1) to determine whether there was a difference in the mediating effect between the two groups. In the high planning group, the mediating effect of self-discipline was 2.906, 95% CI = [2.162, 3.732]; in the low planning group, the mediating effect of self-discipline was 2.862, 95% CI = [2.128, 3.681]; the comparison mediating effect was significant (*p* < 0.01), 95% CI = [0.016, 0.078]. This indicates that the mediation effect is stronger in the higher Planning condition and the mediation model with moderation is validated. Hypothesis 5 is valid.

#### Model 2: Impact on Chinese academic achievement

Referring to the steps of analysis of mediated model with moderation summarized by Wen and Ye (2014) and combined with the hypotheses of this study, Cognitive ability (X), Self-discipline (W), Chinese Academic Achievement (Y), and Planning (U) were used as variables. In addition, all indicators were standardized to reduce the effect of multicollinearity. First, a direct effect model was developed with Cognitive ability(X) as the independent variable and Chinese Academic Achievement (Y) as the dependent variable, and the direct effect was tested to see if it was moderated by Planning(U). The results show that the model without the interaction term fits well: χ^2^/df = 4.63, CFI = 0.914, TLI = 0.867, RMESA = 0.118, and SRMR = 0.056. For the mediated model with the interaction term, AIC = 73863.031, compared to the AIC value of the baseline model (74568.601), which is reduced by 705.570, indicating that the mediated model with adjustment has improved compared with the baseline model; At the same time, For the mediated model with the interaction term, Log Likelihood = –36888.516, which is 360.785 higher than the Log Likelihood of the baseline model (–37249.031), that is, the value of –2LL is 360.785, the degree of freedom of the model is increased by 8, and the chi-square test of –2LL value is significant (*p* < 0.05), so the mediated model with adjustment fits better than the baseline model.

Cognitive ability (X) significantly predicts Chinese Academic Achievement (Y)(β = 0.386, *p* < 0.001), 95% CI = [0.291, 0.471]; the interaction term of Cognitive ability and Planning was a significant predictor of Chinese Academic Achievement (β = 0.099, *p* < 0.05), 95% CI = [0.024, 0.178]. It indicates that the direct relationship between Cognitive ability and Chinese Academic Achievement is moderated by Planning.

**FIGURE 4:**
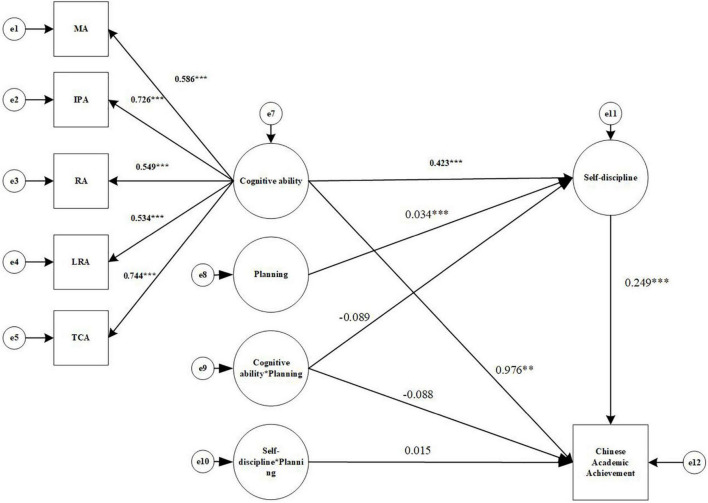
Structural equation modeling results diagram (Chinese Academic Achievement). MA, Memory ability; IPA, Information processing ability; RA, Representation ability; LRA, Logical reasoning ability; TCA, Thinking conversion ability. *p < 0.05, ^**^p < 0.01, ^***^p < 0.001.

Second, the effect of Cognitive ability (X) on Chinese Academic achievement (Y) through Self-discipline (W) was tested to see if it was moderated by Planning (U), and the results are shown in Figure 4. The data show that the model fit index is well: χ^2^/df = 5.01, CFI = 0.906, TLI = 0.854, RMESA = 0.124, and SRMR = 0.060. For the mediated model with adjustment including the interaction term, AIC = 65901.693, compared to the AIC value of the baseline model (74428.485), which is reduced by 8526.792, indicating an improvement of the mediated model with adjustment compared to the baseline model; meanwhile, the Log Likelihood of the mediated model with adjustment = –32907.847, compared to the Log Likelihood of the baseline model (–37178.242), increased by 4270.395, which means that the –2LL value is 4270.395, The increase in the degrees of freedom of the model is 8, and the chi-square test for the –2LL value is significant (*p* < 0.05), so the mediated model with adjustment fits better than the baseline model.

The predictive effect of Cognitive ability (X) on Chinese Academic Achievement (Y) was significant (β = 0.282, *p* < 0.001), 95% CI = [0.176, 0.381]. Cognitive ability (X) can significantly predict Self-discipline (W) (a1 = 0.423, *p* < 0.001), 95% CI = [0.342, 0.497]. the interaction term (UX) of Cognitive ability (X) and Planning (U) cannot significantly predict Self-discipline (W) (a3 = –0.089, *p* = 0.205), 95% CI = [–0.220, 0.052], containing 0. Self-discipline(W) can significantly predict Chinese Academic Achievement(Y) (b1 = 0.249, *p* < 0.001), 95% CI = [0.161, 0.336]; the interaction term (UX) between Cognitive ability (X) and Planning (U) cannot significantly predict Chinese Academic Achievement (Y) (c3′ = –0.088, *p* = 0.052), 95% CI = [–0.178, 0.001], containing 0; the interaction term (UW) between Self-discipline (W) and Planning (U) cannot significantly predict Chinese Academic Achievement (Y)(b2 = 0.015, *p* = 0.637), 95% CI = [–0.049, 0.075], containing 0. In addition, 95% CI for a1b2, a3b1, and a3b2 were calculated using the Bootstrap method and were [–0.003, 0.004], [–85.243, 98.343], and [–1.645, 1.110], respectively, with 95% CI all contained 0. Therefore, the mediating effect of Self-discipline between Cognitive ability and Chinese Academic achievement was significant [mediating effect = 0.343, SE = 0.069, *p* < 0.001, 95% CI = (0.224.0.504)], but the moderating effect of Planning was not significant.

#### Model 3: Impact on Mathematics academic achievement

Referring to the steps of analysis of mediated model with moderation summarized by Wen and Ye (2014) and combined with the hypotheses of this study, Cognitive ability (X), Self-discipline (W), Mathematics Academic achievement (Y), and Planning (U) were used as variables. In addition, all indicators were standardized to reduce the effect of multicollinearity. First, a direct effect model was developed with Cognitive ability(X) as the independent variable and Mathematics Academic achievement(Y) as the dependent variable, and the direct effect was tested to see if it was moderated by Planning(U). The results show that the model without the interaction term fits well: χ^2^/df = 4.72, CFI = 0.909, TLI = 0.858, RMESA = 0.120, and SRMR = 0.059. For the mediated model with the interaction term, AIC = 74066.091, compared to the AIC value of the baseline model (74767.932), which is reduced by 700.901, indicating that the mediated model with adjustment has improved compared with the baseline model; At the same time, For the mediated model with the interaction term, Log Likelihood = –36990.486, which is 358.480 higher than the Log Likelihood of the baseline model (–37348.966), that is, the value of –2LL is 358.480, the degree of freedom of the model is increased by 8, and the chi-square test of –2LL value is significant (*p* < 0.05), so the mediated model with adjustment fits better than the baseline model.

Cognitive ability was a positive predictor of Mathematics Academic achievement (β = 0.278, *p* < 0.001), 95% CI = [0.180, 0.370]; the interaction term of Cognitive ability and Planning was a non-significant predictor of Mathematics Academic achievement was not significantly predicted by the interaction term between Cognitive ability and Planning (β = 0.036, *p* = 0.377), 95% CI = [–0.044, 0.118], containing 0. This indicates that the direct relationship between Cognitive ability and Mathematics Academic achievement was not affected by Planning of the direct relationship was not moderated by Planning.

**FIGURE 5:**
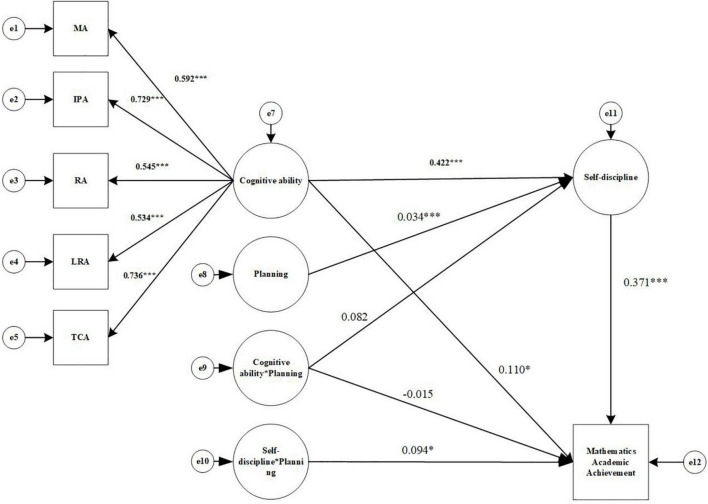
Structural equation modeling results diagram (Mathematics Academic Achievement). MA, Memory ability; IPA, Information processing ability; RA, Representation ability; LRA, Logical reasoning ability; TCA, Thinking conversion ability. *p < 0.05, ^***^p < 0.001.

Self-discipline (W) was tested to see if it was moderated by Planning (U), and the results are shown in Figure 5. The data show that the model fit index is well: χ^2^/df = 5.154, CFI = 0.900, TLI = 0.844, RMESA = 0.126, and SRMR = 0.059. For the mediated model with adjustment including the interaction term, AIC = 66101.190, compared to the AIC value of the baseline model (74628.260), which is reduced by 8527.070, indicating an improvement of the mediated model with adjustment compared to the baseline model; meanwhile, the Log Likelihood of the mediated model with adjustment = –33007.595, compared to the Log Likelihood of the baseline model (–37278.130), increased by 4270.535, which means that the –2LL value is 4270.535, The increase in the degrees of freedom of the model is 8, and the chi-square test for the –2LL value is significant (*p* < 0.05), so the mediated model with adjustment fits better than the baseline model.

Cognitive ability (X) significantly predicts mathematics Academic Achievement (Y) (β = 0.110, *p* < 0.05), 95% CI = [0.002, 0.216]. Cognitive ability (X) can significantly predict Self- discipline (W) (a1 = 0.422, *p* < 0.001), 95% CI = [0.339, 0.497]. The interaction term (UX) of Cognitive ability (X) and Planning (U) cannot significantly predict Self-discipline (W) (a3 = 0.082, *p* = 0.221), 95% CI = [–0.053, 0.210], containing 0. Self-discipline(W) can significantly predict Mathematics Academic achievement(Y) (b1 = 0.371, *p* < 0.001), 95% CI = [0.292, 0.444]; the interaction term (UX) between Cognitive ability (X) and Planning (U) cannot significantly predict Mathematics Academic achievement (Y) (c3′ = –0.015, *p* = 0.725), 95% CI = [–0.100, 0.065]; the interaction term (UW) of Self-discipline (W) and Planning (U) can significantly predict Mathematics Academic achievement (Y) (b2 = 0.094, *p* < 0.05), 95% CI = [0.023, 0.178]. In addition, 95% CI for a1b2, a3b1, and a3b2 were calculated using the Bootstrap method and were [0.002, 0.011], [–0.005, 0.022], and [0.000, 0.000], respectively, with only 95% CI for a1b2 not containing 0. Thus, It is clear that the mediating effect of self-discipline between cognitive ability and academic achievement in mathematics was significant [mediating effect = 0.279, SE = 0.130, *p* < 0.05, 95% CI = (0.001.0.518)], while the discipline’s mediating effect was moderated by planning in the second half, the effect of Self-discipline on Mathematics Academic achievement varied with the level of Planning; the first half of Self-discipline’s mediating effect was not The first half of the mediating effect of Self-discipline was not moderated by Planning.

**FIGURE 6:**
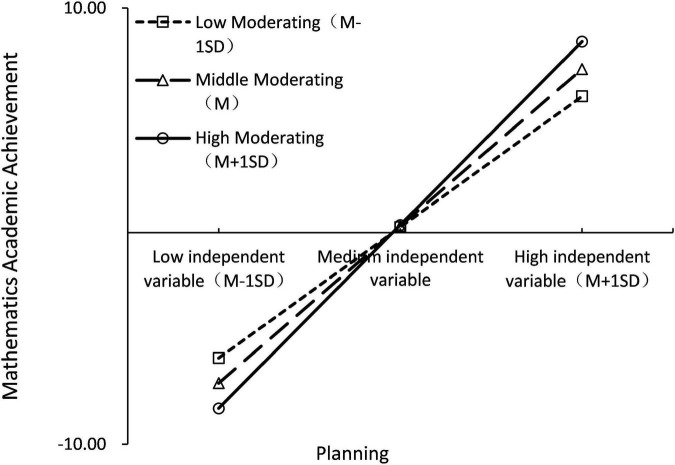
Simple slope test (mathematics academic achievement).

To further explore the moderating effect of Planning, students were divided into “high/low Planning groups” using a positive/negative one standard deviation boundary for Planning. The simple slope test (see Figure 6) showed that the positive predictive effect of Self-discipline on Mathematics Academic achievement was significant in the “high Planning group” (β = 0.793, *p* < 0.001); in the “Low Planning group,” although the predictive effect was still significant (β = 0.775, *p* < 0.001), it was weaker than in the “High Planning group.”

In addition, when the process of mediation is moderated, it is required to test whether the mediating effect varies with changes in the moderating variable U, as suggested by Edwards and Lambert (2007), which is to take the value of one standard deviation above and below the mean of U (standard UH = 1, UL = –1) to determine whether there was a difference in the mediating effect between the two groups. In the high planning group, the mediating effect of self-discipline was 0.282, 95% CI = [0.001, 0.524]; in the low planning group, the mediating effect of self-discipline was 0.276, 95% CI = [0.002, 0.513]; the comparison mediating effect was significant (*p* < 0.05), 95% CI = [0.001, 0.017]. This indicates that the mediation effect is stronger in the higher Planning condition and the mediation model with moderation is validated.

#### Model 4: Impact on English academic achievement

Referring to the steps of analysis of mediated model with moderation summarized by Wen and Ye (2014) and combined with the hypotheses of this study, Cognitive ability (X), Self-discipline (W), English Academic achievement (Y), and Planning (U) were used as variables. In addition, all indicators were standardized to reduce the effect of multicollinearity. First, a direct effect model was developed with Cognitive ability(X) as the independent variable and English Academic achievement(Y) as the dependent variable, and the direct effect was tested to see if it was moderated by Planning(U). The results show that the model without the interaction term fits well: χ^2^/df = 4.64, CFI = 0.911, TLI = 0.861, RMESA = 0.118, and SRMR = 0.058. For the mediated model with the interaction term, AIC = 74024.164, compared to the AIC value of the baseline model (74728.602), which is reduced by 704.438, indicating that the mediated model with adjustment has improved compared with the baseline model; At the same time, For the mediated model with the interaction term, Log Likelihood = –36969.082, which is 360.219 higher than the Log Likelihood of the baseline model (–37329.301), that is, the value of –2LL is 360.219, the degree of freedom of the model is increased by 8, and the chi-square test of –2LL value is significant (*p* < 0.05), so the mediated model with adjustment fits better than the baseline model.

Cognitive ability was a positive predictor of English Academic achievement (β = 0.274, *p* < 0.001), 95% CI = [0.178, 0.363]; the interaction term of Cognitive ability and Planning was a non-significant predictor of English Academic achievement (β = 0.088, *p* < 0.01), 95% CI = [–0.022, 0.175], contains 0. This indicates that the direct relationship between Cognitive ability and English Academic achievement is not moderated by Planning of moderation.

**FIGURE 7:**
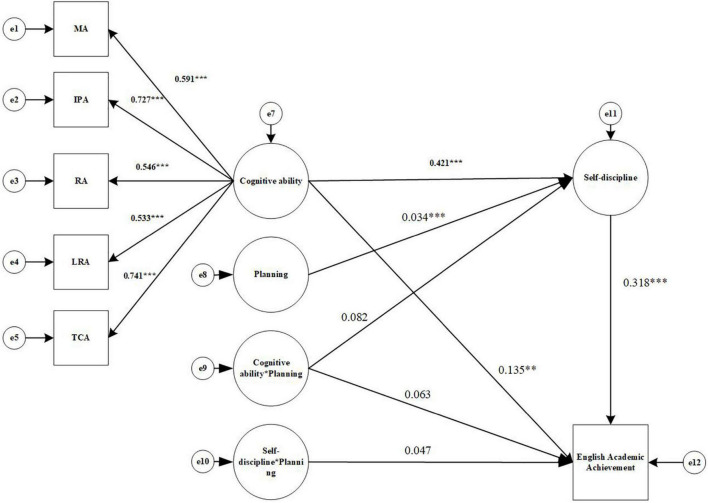
Structural equation modeling results diagram (English Academic Achievement). MA, Memory ability; IPA, Information processing ability; RA, Representation ability; LRA, Logical reasoning ability; TCA, Thinking conversion ability. *p < 0.05, ^***^p < 0.001.

Second, the effect of Cognitive ability (X) on English Academic achievement (Y) through Self-discipline (W) was tested to see if it was moderated by Planning (U), and the results are shown in Figure 7. The data show that the model fit index is well: χ^2^/df = 5.03, CFI = 0.902, TLI = 0.848, RMESA = 0.124, and SRMR = 0.059. For the mediated model with adjustment including the interaction term, AIC = 66061, 794, compared to the AIC value of the baseline model (74588.633), which is reduced by 8526.839, indicating an improvement of the mediated model with adjustment compared to the baseline model; meanwhile, the Log Likelihood of the mediated model with adjustment = –32987.897, compared to the Log Likelihood of the baseline model (–37258.317), increased by 4270.420, which means that the –2LL value is 4270.420, The increase in the degrees of freedom of the model is 8, and the chi-square test for the –2LL value is significant (*p* < 0.05), so the mediated model with adjustment fits better than the baseline model.

Cognitive ability (X) significantly predicts English Academic Achievement (Y) (β = 0.135, *p* < 0.05), 95% CI = [0.027, 0.235]. Cognitive ability (X) can significantly predict Self-discipline (W) (a1 = 0.421, *p* < 0.001), 95% CI = [0.339, 0.496]. The interaction term (UX) of Cognitive ability (X) and Planning (U) cannot significantly predict Self-discipline (W) (a3 = 0.082, *p* = 0.221), 95% CI = [–0.052, 0.209], containing 0. Self-discipline(W) can significantly predict English Academic achievement(Y) (b1 = 0.318, *p* < 0.001), 95%CI = [0.233, 0.397]; the interaction term (UX) between Cognitive ability (X) and Planning (U) cannot significantly predict English Academic achievement (Y) (c3′ = 0.063, *p* = 0.206), 95% CI = [–0.035, 0.164]; the interaction term (UW) between Self-discipline (W) and Planning (U) cannot significantly predict English Academic achievement (Y) (b2 = 0.047, *p* = 0.179), 95% CI = [–0.022, 0.117]. In addition, 95% CI for a1b2, a3b1, and a3b2 were calculated using the Bootstrap method and were [–0.001, 0.011], [–0.004, 0.018], and [0.000, 0.000], respectively, 95% CI containing 0. Thus, it can be seen that Self-discipline in Cognitive ability and English Academic achievement was significant [mediating effect = 0.266, *SE* = 0.096, *p* < 0.01, 95% CI = (0.068.0.452)], but the moderating effect of Planning was not significant.

## Discussion

### The direct impact of cognitive ability

In China, the high school level is the best time for students to develop their cognitive abilities, and the intensive learning difficulty and frequent examinations also help students to further improve their cognitive abilities while demonstrating the important role of cognitive abilities in academic achievement. At the high school level, the impact of different types of cognitive abilities on Academic Achievement varies across subjects (Zuo and Wang, 2016; Liang et al., 2020).

IPA (information processing ability) mainly refers to students’ ability to comprehend information after obtaining it through reading and listening, and is closely related to students’ classroom efficiency. Students with higher IPA ability can fully understand and master what teachers teach in class and quickly construct their own knowledge system, thus improving their knowledge mastery and achieving better academic achievement in exams (Yuan and Wen, 2003). The effects of IPA on academic achievement in Chinese and mathematics are comparable because, under the current testing system in China, mathematics exams increase students’ ability to read and extract key information and focus more on their thinking skills, while Chinese exams also increase the amount of reading and require students to have a high IPA to achieve better academic achievement (Lin, 2021). The impact of IPA on academic achievement in English is weaker than that in Chinese and mathematics because the current test focuses on the vocabulary range of students in English, and less on the deeper reading and thinking skills than in Chinese.

TCA (Thinking Conversion Ability) refers to the student’s active thinking in the learning process to understand knowledge, mainly in the speed and accuracy of thinking conversion, therefore, this ability can facilitate learning in various disciplines, especially in mathematics, where students with high TCA ability can more easily summarize existing knowledge and expand new thinking and skills in the problem solving process, and to make analogies in similar problems, thus improving the accuracy of solving novel and difficult problems, and thus improving their academic achievement (Liu, 1988).

MA (memory ability) mainly refers to students’ long term memory ability. the stronger the MA ability value, the more students can remember knowledge quickly and retain it over time (Liang et al., 2020). In addition, MA can interact with IPA to grasp more reading information through memory ability, which leads to faster comprehension of information during reading and significantly improves students’ reading ability, which is especially evident in reading comprehension questions in English and Chinese exams. As a result, academic achievement is also better among students with stronger MA proficiency values (Yu et al., 2014).

LRA (Logical Reasoning Ability) includes both inductive and deductive reasoning types. Recently, higher education entrance examinations in China have begun to focus on students’ reasoning ability, which is also reflected in the addition of a large number of logical reasoning questions in Chinese and English subjects (Hu, 2017); therefore, LRA also has a significant positive impact on academic achievement in Chinese and English examinations.

RA (representational ability) is a key cognitive ability that can effectively help students understand mathematical spatial imagery-like knowledge content (Shao et al., 2004). Also, RA can stimulate associative memory by forming images in students’ minds during the process of reciting knowledge related to Chinese and English, thus making students’ memory of knowledge stronger and longer lasting, and thus improving academic achievement (Lin et al., 2003).

### The mediating role of self-discipline

The present study showed that self-discipline partially mediates the relationship between cognitive ability and Academic Achievement. Self-discipline is a behavioral habit, and self-management is the essence of self-discipline. Self-discipline follows certain principles and norms and relies on the student’s own willpower to accomplish the desired goal’s behavioral ability without supervision (Liu, 2020).

A higher level of self-discipline means that students are more able to concentrate and resist external temptations during the learning process, which can help them be more effective in their studies. Especially for high school students, the higher the value of self-discipline, the better they can make the most of their time, the more efficient and independent thinking they can do, thus improving their understanding and mastery of knowledge and leading to improved academic achievement. In addition, according to the teaching schedule in Beijing, school ends at 16:30 every afternoon and students manage the rest of the day by themselves. Students with high self-discipline can fully and rationally use their time, thus consolidating what they have learned, improving their understanding and mastery, and achieving better overall academic results in academic exams.

The stronger the cognitive ability of students, the more efficient students are in their studies, the more focused they are in their studies, and the more likely they are to achieve a sense of academic achievement (Wang, 2011). Therefore, the stronger the cognitive ability of students, the easier it is to immerse themselves in learning, the more willing they are to learn actively and positively, and the more able they are to customer service external temptations, thus continuously improving their self-discipline. When students encounter difficulties and challenges in learning, students with strong cognitive ability have stronger creative thinking ability, such as TCA, RA, and LRA, which can help students solve problems more quickly and efficiently, thus allowing students to stay focused on learning and develop strong self-discipline (Wo and Lin, 2000); when students’ learning is disturbed by the outside world, students with strong cognitive ability This helps students to resist loose learning behaviors and develop stronger self-discipline, so that they can devote more time and energy to their studies, improve their learning efficiency, and enhance their academic achievement (Zheng and Zhang, 2021).

Specifically, self-discipline plays a partially mediating role in overall academic achievement, with a mediating role of 0.263 and a mediating effect of 38.3%; in Chinese academic achievement, self-discipline plays a partially mediating role, with a mediating role of 0.105 and a mediating effect of 27.1%; in mathematics academic achievement, self-discipline plays a partially mediating role, with a mediating role of 0.157 and a mediating effect of The mediating effect of self-discipline in English academic achievement was partially mediated by 0.134, with a mediating effect of 49.8%. As can be seen, the mediating effect of self-discipline is more pronounced in mathematics and English subjects, which is because the examinations of mathematics subjects pay more attention to the assessment of students’ thinking ability, which requires students to think and train for a long time, especially in TCA and LRA, which require students to improve and show these abilities in a long time and highly focused learning process, so for students with strong cognitive ability, the therefore, for students with strong cognitive abilities, the stronger their self-discipline and the longer they focus during the examinations, the more they can show the characteristics of their TCA and LRA abilities, and the more they can achieve better academic results during the examinations. In addition, in the English subject test subjects, the effect of the mediating role of self-discipline in multiple English subjects also appears to be prominent as the test questions become increasingly important in assessing students’ thinking over and above the assessment of mastery of basic knowledge (Lin, 2021) with the advancement of Chinese test reform.

### The moderating mediating role of planning

The present study found that planning moderates the second half of academic achievement by influencing academic achievement. Specifically, students with high plannings were more likely to affect academic achievement through cognitive ability compared to students with low plannings, and hypotheses 4 and 5 were tested. Furthermore, in contrast to our expectations, planning did not moderate the direct effect of cognitive ability and the first half of the mediating effect.

The moderating effect of planning was significant in the second half of the mediating effect. planning is an important component of an individual’s self-regulated learning strategy, and as a personality trait, it can stimulate factors such as an individual’s motivation to learn and the ability to self-regulate learning activities, thus facilitating the accomplishment of learning goals (Cao and Cao, 2004), and students who perceive themselves as having a better command of time In addition, the stronger a student’s planning is, the better the student’s ability to manage time, which is a guarantee of high learning effectiveness and high academic achievement (Wang, 2003).

In the first half of the mediating effect, the moderating effect of planning is not significant. The reason for this is that in the stage of influence of cognitive ability on self-discipline, it is mainly through the sense of academic achievement and academic self-confidence built up by cognitive ability that positively influences self-discipline and thus obtains better positive feedback, and this sense of academic achievement does not change with the adjustment of academic plan, so planning is not significant in regulating the influence of cognitive ability on self-discipline.

Also, among the subject-specific subjects, the moderating effect of planning was significant among academic achievement in mathematics, but not in Chinese and English. This is because for mathematics subjects, learning requires more rigorous logical arrangement and reasonable time security in order to fully understand and digest the knowledge learned and to improve one’s thinking ability, so as to improve academic achievement in mathematics. In contrast, Chinese and English subjects are more important to the accumulation of daily knowledge, which requires students to persist for a long time to see the effect, which is not related to the planning of learning arrangements (Lin, 2021), so the moderating effect of planning is not significant.

In addition, the simple slope test revealed that when self-discipline is low, students with low planning ability perform better than students with high planning ability; when self-regulation is high, students with high planning ability perform better than students with low planning ability. This is due to the fact students with weak self-discipline, the stronger their planning ability and the more reasonable their time planning, the more likely they are to put pressure on themselves, which leads to weak execution ability and the inability to complete the planned study tasks, thus leading to lower and lower academic achievement. In contrast, when self-discipline is high, the stronger the planning ability and the more reasonable the time planning arranged, the more it helps students to improve their learning efficiency and give full play to their cognitive ability, thus excelling in academic achievement.

### Limitations and future directions

In this study, only the external influences caused by two of the personality traits, self-discipline and planning, were considered in the analysis of the influence of cognitive ability on sustained academic achievement; similarly, the influence of other psychological states of the students on cognitive ability and academic achievement was not considered in the analysis. In addition, one of the most prominent limitations of this study is the small sample size and the single range of students investigated. To further enhance the credibility of the study results, more schools in other Chinese provinces should be selected for study and comparison. Future research could focus on this area to obtain more valuable findings.

## Conclusion

This study used structural equation modeling to analyze the moderating effect of panning on the mediating effect of self-discipline on the relationship between cognitive ability and academic achievement. The results showed that cognitive ability can have a significant positive effect on academic achievement, while self-discipline plays a partially mediating role between cognitive ability and academic achievement, and the moderating effect of Planning is significant in the second half of the mediating effect, i.e., the effect of self-discipline on academic achievement changes as the level of planning increases, and the mediating effect is stronger in the condition of higher planning, and the mediating model with moderating effect holds.

## Data availability statement

The original contributions presented in this study are included in the article/Supplementary material, further inquiries can be directed to the corresponding author.

## Ethics statement

The studies involving human participants were reviewed and approved by the Research Ethics Committee of the School of Humanities and Social Sciences, University of Science and Technology Beijing. Written informed consent to participate in this study was provided by the participants or their legal guardian/next of kin.

## Author contributions

YS contributed to the conception and design of the study and performed the statistical analysis. YS and SQ contributed to the data collection and wrote the first draft of the manuscript. Both authors contributed to the manuscript revision, read, and approved the submitted version.
